# Mini-review: The health benefits and applications of allicin

**DOI:** 10.3389/fphar.2025.1715922

**Published:** 2025-11-26

**Authors:** Ke-Qian Chen, Hai-Bo Lei, Xiang Liu, Wen-Jing Cao

**Affiliations:** 1 Department of Clinical Pharmacy, Xiangtan Central Hospital (The Affiliated Hospital of Hunan University), Xiangtan, China

**Keywords:** allicin, garlic, health benefit, application, herb

## Abstract

Garlic is a perennial plant with a long history of cultivation and medicinal use. As a valuable food additive and spice, garlic contains many nutrients and chemicals. Allicin is a key bioactive organosulfur metabolite derived from garlic bulbs. In recent years, more and more scientists have carried out research on it. This article reviews the health benefits and applications of allicin. Meanwhile, we also discussed the limitations of relevant research on allicin and look forward to the future prospects of allicin. We found that allicin is a valuable metabolite in medicine and the food field. With the deepening of allicin research, the application of allicin will have a broader prospect.

## Introduction

Garlic, *Allium sativum* L. (Amaryllidaceae), is a perennial plant with a long history. It is documented that garlic has been cultivated in Mediterranean countries since 3000 BC. As a valuable food additive and spice, garlic contains many nutrients and chemicals. These substances mainly include sulfur compounds, volatile oils, amino acids, and glycosides. Allicin is a metabolite isolated from garlic bulbs. In 1944, Cavallito first isolated and described the properties of allicin (Cavallito and Bailey, 1944). In 1948, Stoll determined the structure of allicin (Stoll and Seebeck, 1948). Interestingly, fresh garlic does not contain allicin. Studies have shown that fresh garlic contains the precursor of allicin (alliin). When garlic is sliced or mashed, alliin and alliinase, which are distributed in different parts of cells, combine to form allicin (Deng et al., 2023). The properties of allicin are unstable, and it can be rapidly transformed into Allyl methyl sulfide (AMS), Diallyl trisulfide (DATS), and Diallyl disulfide (DADS) by heating, extrusion, or organic solvent treatment. These metabolites are sensitive to organic solvents, oxygen, temperature, and pH (Deng et al., 2023). TRPA1 and TRPV1, two temperature-activated ion channels, are present in the pain-sensing neurons that innervate the mouth. Macpherson LJ et al. found that allicin causes the pungency of garlic by activating transient receptor potential cation channel subfamily A member 1 (TRPA1) and transient receptor potential cation channel subfamily V member 1 (TRPV1) (Macpherson et al., 2005). As an important alkaloid, it is of great significance to understand the properties of allicin. Allicin generally has a strong odor (Borlinghaus et al., 2021). Its chemical name is diallyl thiosulfinate (Hirata et al., 2025). Allicin is soluble in benzene, ethyl alcohol, ether, and other organic solvents (Hirata et al., 2025). In addition, another key property of allicin is its hydrophobicity, which allows it to be absorbed easily through the cell membrane without causing any physical or chemical damage to the phospholipid bilayer, thereby allowing its rapid metabolism to produce pharmacological effects (Xu S. et al., 2023). In general, allicin is unstable in alkaline environments, but stable in acidic environments (Fujisawa et al., 2008). In recent years, with the clinical application of allicin, more and more scientists have carried out research on allicin. Allicin exhibits protective effects in multiple organ systems, including the brain, intestines, lungs, liver, kidneys, prostate, and heart. This article reviews the health benefits and applications of allicin. Meanwhile, we also discussed the limitations of relevant research on allicin and look forward to the future prospects of allicin.

## Pharmacokinetics of allicin

In recent years, drug delivery systems that enhance the stability of allicin have become a hot topic. Therefore, it is very important to understand the pharmacokinetics of allicin. Previous reports revealed that allicin has a short half-life and a large first-pass effect (Bhuker et al., 2024). The liver is the main organ of the metabolism of allicin. Meanwhile, reduction, methylation, and oxidation are the main metabolic reactions of allicin. Allicin is rapidly decomposed in the liver to form a variety of organic sulfides, including S-allylmercaptoglutathione, S-allylmercaptocysteine, allyl methyl sulfide, and allyl mercaptan (Egen-Schwind et al., 1992). Because allicin is eliminated from the body by the respiratory tract, the concentration of allicin in lung tissue is significantly lower than that in the blood (Zhang et al., 2020). Lachmann G. et al. studied the pharmacokinetics of allicin in rats using ^35^S isotope labeling technique (Lachmann et al., 1994). They found that the peak time (Tmax) of allicin was 30min–60 min after oral administration. According to fecal excretion data and urinary excretion data, the overall excretion of allicin was 85.5% after 72 h. Meanwhile, the minimum absorption rate of allicin was 65% after 72 h. Li M et al. studied the pharmacokinetic parameters of allicin in rabbits by intravenous injection. They found that the main pharmacokinetic parameters were as follows: half-life (t_1/2_)of allicin was 227 min–260 min, the area under the curve (AUC) of allicin was 12583.1 (mg min)/L, the apparent volume of distribution was 4.51 L, and the clearance of allicin was 0.012 mg/(L min) (Lachmann et al., 1994). We believe that the bioavailability of allicin is relatively low for the following reasons: At first, allicin is characterized by a distinctive garlic odor and chemical instability. It can be easily degraded under room temperature. Secondly, alliin is converted to allicin under the action of allinase. Allinase can be inhibited in the stomach due to its sensitivity to gastric acid. Therefore, enteric-coated preparations have been developed to prevent the degradation of allinase in the stomach (Lawson and Hunsaker, 2018). Thirdly, the bioavailability of allicin is highly influenced by its formula. There were significant differences in the bioavailability of allicin in healthy subjects after eating boiled garlic food, roasted garlic food, pickled garlic food, and garlic powder capsules for 32 h (Bala et al., 2024). Although these characteristics of allicin limit its clinical potential, it can easily cross cell membranes to exert pharmacological activities due to its lipophilic properties and hydrophobic properties. Overall, it is of great significance to explore the pharmacokinetics of allicin for application and improvement of allicin in the future.

## Health benefits of allicin

### Antimicrobial activity

As a common clinical disease, infectious disease is usually caused by pathogenic microorganisms. Pathogenic microorganisms encompass a wide range of infectious agents, including bacteria, fungi, viruses, and parasites. Inhibiting and killing these pathogenic microorganisms is the best strategy to prevent and treat infectious diseases. Numerous studies have shown that allicin is a strong natural antimicrobial substance. However, the majority of this evidence is derived from *in vitro* studies, and its translation to clinical settings requires careful evaluation of the effective concentrations achieved *in vivo*. Allicin can inhibit the growth of *Mycobacterium tuberculosis* (Dwivedi et al., 2019)*, Escherichia coli* (Chang et al., 2022)*, Trichosporon asahii* (Yang et al., 2023)*, Pseudomonas aeruginosa* (Xu et al., 2019)*, Salmonella Typhimurium* (Feldberg et al., 1988), *Trichophyton rubrum* (Aala et al. 2012), *Staphylococcus aureus* (Leng et al., 2011)*, Helicobacter pylori* (Si et al., 2019)*, Candida albicans* (Khodavandi et al., 2011)*, Cryptococcus neoformans* (Li Z. et al., 2022), and *mucorales* (Schier et al., 2023). The minimum inhibitory concentration of allicin against different bacteria varied greatly. The neutral and alkaline environment is conducive to the antibacterial action of allicin. High temperatures can accelerate the decomposition of allicin and reduce the antibacterial effect. The mechanism research showed that the antibacterial effect of allicin was linked to its sulfydryl. Sulfydryl can react with alcohol dehydrogenase, thioredoxin reductase, and RNA polymerase to affect the essential metabolism of cysteine proteinase (Wang JR. et al., 2022). Meanwhile, allicin also plays an antimicrobial role by inhibiting biofilm formation (Khodavandi et al., 2011), DNA gyrase (Reiter et al., 2020), Cu^2+^/Zn^2+^ uptake (Prescott and Panaretou, 2017), and virulence factors’ production (Lihua et al., 2013). While these mechanistic insights are valuable, many are based on cell-free or simple cellular assays, and their relative contribution to the overall antimicrobial effect in complex biological systems warrants further investigation. In addition, allicin can also enhance the bactericidal activity of many antibacterial drugs, including amphotericin B (An et al., 2009), polymyxin B (Ogita et al., 2007), Norfloxacin (Alam et al., 2018), cefoperazone (Cai et al., 2007), cefazolin (Cai et al., 2007), and vancomycin (Zhai et al., 2014). These synergistic studies, often conducted *in vitro*, highlight a promising therapeutic strategy but need validation in animal infection models to assess their clinical potential. High-performance liquid chromatography, mass spectrometry, photodiode array detection, and microdilution are often used to detect the antibacterial properties of allicin (Pérez-Giraldo et al., 2003; Phan et al., 2019). Besides its antibacterial properties, allicin has been found to possess antiviral effects against a range of viruses, including *Human herpesvirus 1, Human herpesvirus 2, Human parainfluenza virus 3, Human rhinovirus B, Vesicular stomatitis virus, Vaccinia virus, Coxsackievirus, and Gammaretrovirus* (Luo et al., 2009). Different preparations also affect the antimicrobial effects of allicin. Dong Qing Luo et al. found that polybutylcyanoacrylate nanoparticles loaded with allicin have stronger antifungal efficacy than pure allicin (Luo et al., 2009). Future efforts to optimize the formulation and delivery of allicin could significantly enhance its efficacy in treating infectious diseases.

### Hepatoprotective activity

**FIGURE 1:**
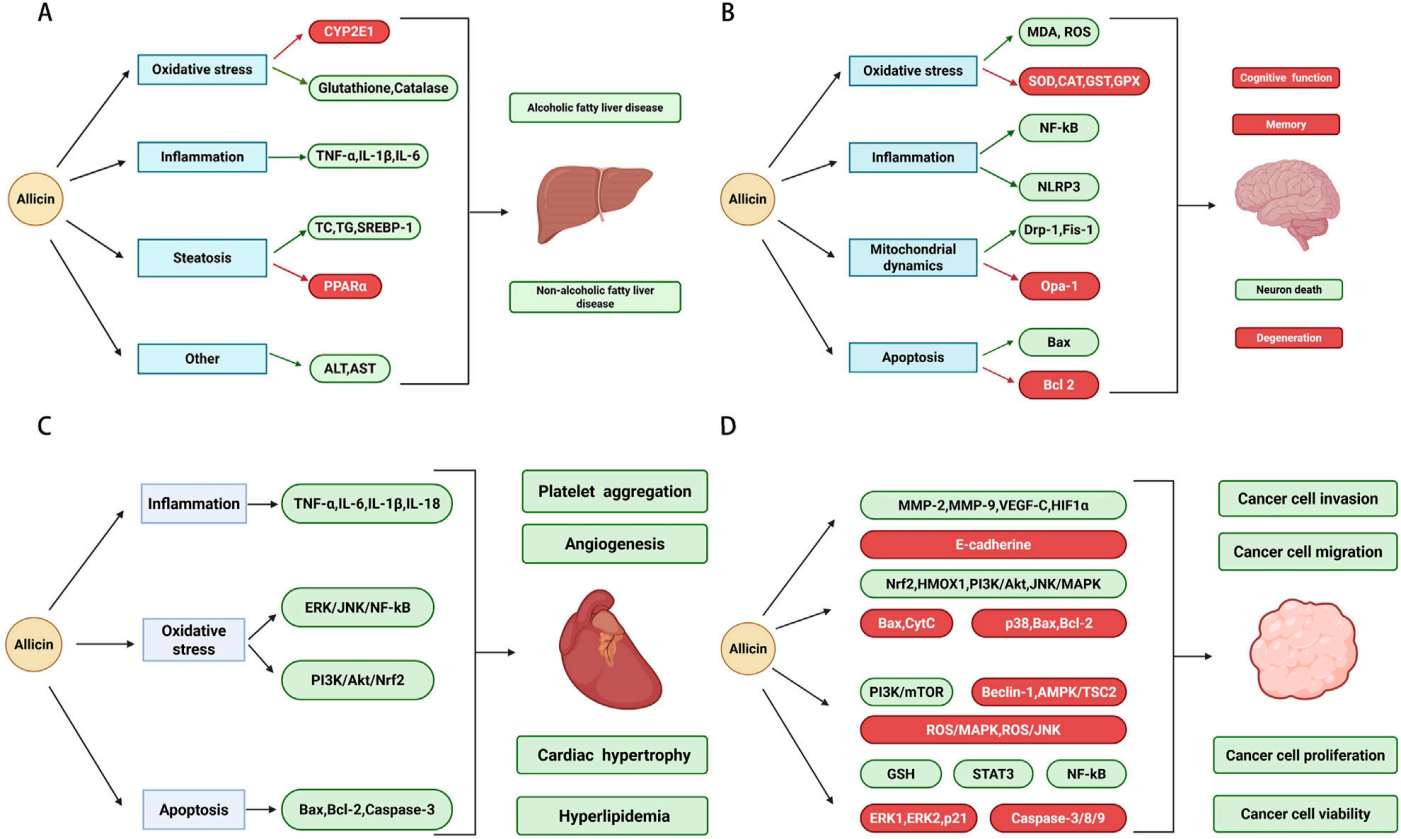
**(A)** The hepatoprotective activity of allicin. **(B)** The neuroprotective activity of allicin. **(C)** The cardioprotective activity of allicin. **(D)** The anticancer activity of allicin.

As a complex pathological process, lipid metabolism disorder is the initial stage of fatty liver disease. Lin XL et al. investigated the effects of allicin on foam cells (Lin et al., 2017). They found that pre-treatment of the foam cells with allicin decreased lipid accumulation (total cholesterol, free cholesterol, and cholesterol ester levels) in cells. Meanwhile, allicin-induced upregulation of ATP binding cassette transporter A1 (ABCA1) promotes cholesterol efflux via Peroxisome proliferator-activated receptor γ (PPARγ)/liver X receptor α (LXRα) signaling in foam cells. Cheng B et al. investigated the protein targets of allicin on lipid metabolism (Cheng et al., 2021). They found that allicin not only upregulated the expression of Peroxisome proliferator-activated receptor α (PPARα) and Fatty acid-binding protein 6 (FABP6) but also downregulated the expression of Fatty acid-binding protein 4 (FABP4) and PPARγ. In 1,3-Dichloro-2-propanol-induced HepG2 cells, allicin alleviated lipid accumulation and lipid metabolism disorder by regulating AMP-activated protein kinase (AMPK)/Sterol regulatory element-binding protein (SREBPs) signaling pathway and Protein kinase A (PKA)/cAMP-response element binding protein (CREB) signaling pathway (Lu et al., 2017). In D-galactosamine/lipopolysaccharide-induced hepatitis rats, allicin increased liver antioxidant enzyme levels and decreased lipid peroxidation (Vimal and Devaki, 2004). From steatosis to fibrosis and cirrhosis, oxidative stress and inflammation are involved in this pathological progression. In alcoholic fatty liver disease (AFLD) mice, allicin increased the levels of glutathione and catalase. Furthermore, allicin decreased the levels of Tumor necrosis factor-α (TNF-α), Interleukin-1β (IL-1β), and Interleukin-6 (IL-6) and inhibited the expression of Cytochrome P450 family 2 subfamily E member 1 (CYP2E1) and Sterol regulatory element-binding protein-1 (SREBP-1) (Panyod et al., 2016). In primary hepatocytes isolated from Sprague-Dawley rats, 10 μM allicin enhances the antioxidation and detoxification capabilities (Wu et al., 2012). By inhibiting oxidative stress and inflammation, allicin plays a hepatoprotective role in tetrachloride-induced mice, trioxide-induced rats, and lead-induced chicken (Cai et al., 2021; Gong et al., 2024; Yang et al., 2017). Liver disease is closely related to gut microbiota. The progression and therapeutic effect of liver disease can be influenced by the regulation of composition and function of the gut microbiota. Studies have shown that allicin reduces inflammation and fat deposition of the liver by regulating gut microbiota (Panyod et al., 2020; Shi et al., 2019). Hepatic ischemia/reperfusion injury is a significant cause of morbidity and mortality following liver surgery. Allicin protects against hepatic ischemia/reperfusion injury via PPARγ/Interleukin-1 receptor-associated kinase-M (IRAK-M)/Toll-like receptor 4 (TLR4) signaling pathway (Li W. et al., 2022). Chemotherapy-induced liver damage has been a common problem during cancer treatment. As an adjuvant to cyclophosphamide and tamoxifen, allicin plays a beneficial role by alleviating liver injury (Suddek, 2014; Sun D. et al., 2021). Meanwhile, some studies have reported that allicin can also improve the Nonsteroidal anti-inflammatory drugs (acetaminophen, diclofenac sodium) -induced liver injury (Orabi et al., 2020; Samra et al., 2020) (Figure 1A). While these pre-clinical findings are promising, they are based on specific, induced pathologies, and the preventive or therapeutic window in humans remains undefined. For example, most evidence comes from rodent models and cell lines, and the doses used in animals must be critically evaluated for their human relevance. The use of a cancer cell line to model metabolic disease is a limitation, as the results may not fully replicate physiology in normal hepatocytes.

### Neuroprotective activity

Over the past few decades, more and more neurological diseases have resulted in premature death or disability as the population ages. As a promising neuroprotective agent, allicin plays a protective role in ischemic stroke (Zhang B. et al., 2015), intracerebral hemorrhage (Atef et al., 2023), subarachnoid hemorrhage (Shao et al., 2019), brain ischemia-reperfusion (Kong et al., 2017), spinal cord ischemia-reperfusion (Zhu et al., 2012), and spinal cord injury (Lv et al., 2015) (Figure 1B). On the one hand, allicin promotes functional recovery via Src tyrosine-protein kinase (SRC)/Protein kinase B (AKT)/Extracellular signal-regulated kinase (ERK) signaling pathway (Zhuang et al., 2023), Heat shock protein 70 (HSP70)/AKT/Inducible nitric oxide synthase (iNOS) signaling pathway (Wang and Ren, 2016), and improvement of mitochondrial function (Zhu et al., 2012). On the other hand, allicin improves cognitive deficits via Protein kinase R-like endoplasmic reticulum kinase (PERK)/Nuclear factor erythroid-2-related factor 2 (NRF2) signaling pathway and c-Jun N-terminal kinase (JNK) signaling pathways (Zhang et al., 2018; Zhu et al., 2015). Alzheimer’s disease (AD) is the most common health problem in aging societies. Oxidative stress, neuroinflammation, and β-amyloid (Aβ) plaque formation are crucial factors in its pathogenesis (Tedeschi et al., 2022). Allicin can ameliorate cognitive dysfunction and reduce neuronal death by inhibiting these pathways in AD models (Li et al., 2010). As the second most common neurodegenerative disease, Parkinson’s disease (PD) has also become a main global health burden. Liu H et al. want to investigate the protective role of allicin in PD. They found that allicin suppressed ROS generation and decreased lipid peroxidation in 6-hydroxydopamine (6-OHDA)-induced Pheochromocytoma 12 (PC12) cells (Liu et al., 2015). Cholesterol homeostasis is essential for the function of the brain. In animal models and cell models, allicin increased cholesterol turnover by up-regulating the expression of ABCA1, 3-hydroxy-3-methylglutaryl coenzyme A reductase (HMGCR), and Cytochrome P450 family 46 subfamily A member 1 (CYP46A1) (Nazeri et al., 2021). Metal ions are essential substances in the human central nervous system. These ions play an important role by participating in various physiological activities in the nervous system. However, some studies have pointed out that metal ions homeostasis imbalance can lead to learning and memory impairment, cognitive impairment, anxiety, and depression. Therefore, it is very meaningful to find a method to treat metal ion poisoning. Cai S found that allicin can ameliorate lead-induced cognitive dysfunction in rats (Cai J. et al., 2019). Kaur S et al. found that allicin can alleviate aluminium-induced cognitive dysfunction and copper-induced cognitive dysfunction in rats (Kaur et al., 2021). In addition to metal ions, allicin also alleviates tunicamycin-induced cognitive dysfunction in rats (Xiang et al., 2017). In conclusion, allicin may be a promising neuroprotective agent.

It is important to emphasize that these findings are pre-clinical, and the blood-brain barrier permeability of allicin and its active metabolites in humans is not fully established. Although these models simulate specific toxicological insults, the applicability of allicin for treating chronic, multifactorial human neurodegenerative conditions requires direct clinical investigation.

### Cardioprotective activity

It is well known that cardiovascular disease is a complex and multifactorial disease. Risk factors such as diabetes, high cholesterol, overweight, and obesity can exacerbate the progression and occurrence of cardiovascular disease. Numerous studies have shown that allicin exerts a protective effect in many cardiovascular diseases, including atherosclerosis (Panyod et al., 2022), hypertension (Cui et al., 2020), myocardial infarction (MI) (Xu et al., 2020), myocardial ischemia–reperfusion (Gao et al., 2021), arrhythmia (Han et al., 2019), and cardiac hypertrophy (Ba et al., 2019) (Figure 1C). However, the evidence is almost exclusively derived from animal models, and the translation to human cardiovascular health is speculative without clinical trial data. As the main component of blood vessels, the activation of vascular endothelial cells can promote angiogenesis. Allicin has been found to protect against myocardial ischemia–reperfusion by accelerating angiogenesis (Liu et al., 2021). Its mechanism involves the inhibition of vascular endothelial cell activity and the activation of miR-19a-3p/Phosphatidylinositide 3-kinases (PI3K)/AKT signaling pathway (Wang Q. et al., 2022). As a potential antioxidant, allicin can protect the cardiovascular system by decreasing the level of Reactive oxygen species (ROS) and stimulating the level of glutathione (Horev-Azaria et al., 2009). For instance, Chen Liu et al. found that allicin protects against cardiac hypertrophy by inhibiting ROS-dependent signaling pathways (Liu et al., 2010). Xian Hui Li et al. found that allicin ameliorates cardiac hypertrophy by enhancing the NRF2 antioxidant signaling pathways (Li et al., 2012a). Another study suggests that allicin ameliorates myocardial infarction by inhibiting p-PERK-mediated oxidative stress (Gao et al., 2024). In the treatment of atherosclerosis and hypertension, allicin exhibits potential through regulation of gut microbiota and induction of vasorelaxation (Cui et al., 2020; Panyod et al., 2022). Meanwhile, allicin also exhibits an antiarrhythmic effect on rats, and the mechanism is related to the inhibition of l-type calcium channels and transient outward potassium current (Cao et al., 2016; Han et al., 2019). Interestingly, allicin has ameliorating effects on cardiotoxicity caused by many drugs, including streptozotocin (Huang et al., 2013), methotrexate (Aboubakr et al., 2023), trastuzumab (Mousa et al., 2022), and doxorubicin (Abdel-Daim et al., 2017). In conclusion, allicin has a promising prospect in the treatment of cardiovascular diseases. While these mechanistic studies are valuable, the doses and routes of administration used in many animal studies are not directly translatable to oral supplementation in humans.

### Anticancer activity

As a leading cause of death globally, cancer has become the biggest public health problem. Conventional cancer therapy methods such as radiation, chemotherapy, immunotherapy, and surgical intervention, were expensive and have side effects. More and more researchers are focusing on natural products for the treatment of cancer. Because these natural products have the advantages of low toxicity and cheap. Numerous studies have shown the benefits of allicin on many cancers, including liver cancer (Chu et al., 2012), gastric cancer (Zhang X. et al., 2015), brain cancer (Cha et al., 2012), lung cancer (Huang H. et al., 2017), bone cancer (Xie et al., 2024), breast cancer (Maitisha et al., 2021), skin cancer (Jobani et al., 2018), cervical cancer (Yifan et al., 2024), ovarian cancer (Xu et al., 2014), thyroid cancer (Xiang et al., 2018), and colon cancer (Huang WL. et al., 2020) (Figure 1D). Genomic instability is a characteristic of carcinogenic process, and the occurrence of all tumors is caused by abnormal DNA damage. Allicin not only directly protects DNA, but also indirectly protects DNA through antioxidant activity and regulation of oxidizing enzymes (Catanzaro et al., 2022). As a defense mechanism, inflammation also plays an important role in the carcinogenic process. During the inflammatory process, many cytokines and chemokines (TNF-α, IL-1β) can stimulate the abundant production of ROS. This process may increase the risk of DNA damage and carcinogenesis. R. Bruck et al. found that allicin inhibited the release of these pro-inflammatory factors (Bruck et al., 2005). In addition to the above mentioned mechanisms, allicin and its secondary metabolites can also inhibit the proliferation, migration, and invasion of these tumor cells by inducing apoptosis and autophagy, increasing the tumor suppressor genes, decreasing angiogenesis, and regulating various pathways. Meanwhile, allicin can overcome doxorubicin resistance, cisplatin resistance, and 5-fluorouracil resistance (Pandey et al., 2020; Shi et al., 2024; Zou et al., 2016). Allicin combined with other chemotherapy drugs showed a better anti-cancer effect (Gao et al., 2015; Jiang et al., 2013). Based on the good tumor suppressor effect and high biosafety, allicin can be used for food adjuvant therapy of chemotherapy. It is worth noting that the results of some experiments are controversial. For example, some studies of allicin and gastric cancer are inconsistent (Kim et al., 2018; Li WQ. et al., 2019). Therefore, it is still important to explore the mechanism and progress of allicin in cancer. However, it is crucial to interpret these findings with caution. A critical caveat is that most evidence is from *in vitro* studies, often employing high concentrations that may not be physiologically achievable *in vivo* through dietary intake or supplementation. The high reactivity of allicin can lead to non-specific cytotoxicity at elevated concentrations, which may not represent a targeted anticancer effect. Furthermore, there are inconsistencies in the literature; for example, findings on allicin’s effects in gastric cancer models are not unanimous (Kim et al., 2018; Li X. et al., 2019), highlighting the need for more standardized research and independent replication.

### Other activity

In addition to the above mentioned health benefits, allicin also has other health benefits (Table.1). Xiao Jun Li found that allicin can ameliorate kidney injury via the NRF2/Heme oxygenase-1 (HO-1) signaling pathway (Li et al., 2024). Besides the NRF2/HO-1 signaling pathway, allicin can also attenuate kidney injury by attenuating oxidative stress, lipid peroxidation, inflammation, and apoptosis (Shan et al., 2021; Xu N. et al., 2023). Xu Dong Wang and Yu Qin Qian found that allicin can attenuate the progression of acute lung injury and osteoarthritis via the PI3K/AKT signaling pathway (Qian et al., 2018; Wang et al., 2018). As a potential therapeutic agent for diabetes, allicin can improve diabetes by inhibiting the formation of advanced glycation end products and accelerating wound healing (Li L. et al., 2022; Toygar et al., 2020). Several studies have investigated the effects of allicin on serum lipids and blood pressure in hypercholesterolemic mice, rats, and rabbits. The results demonstrate that allicin has the potential to ameliorate hypercholesterolemia by reducing blood cholesterol, triglycerides, and cholesterol (Lu et al., 2012; Nawaka et al., 2022). In addition, allicin also plays a role in the treatment of malaria (Coppi et al., 2006), hemorrhagic shock (Zhang Y. et al., 2008), mouth ulcers (Jiang et al., 2015), and psoriasis (Zhang et al., 2023).

**TABLE 1:** Health benefits of allicin.

Health benefit	Type	Model	Concentration or dose	Effects	Ref.
Antimicrobial activity	Bacteria	*S. aureus, Staphylococcus epidermidis* and *P. aeruginosa*	1.0–1,024 mg/L	*S. aureus, Staphylococcus epidermidis* and *P. aeruginosa* ↓	Cai et al. (2007)
	Fungus	*C. albicans*	1–1,024 μg/L	*C. albicans* ↓	An et al. (2009)
	Fungus	*C. albicans*	8–512 μg/mL	*C. albicans* ↓	Guo et al. (2010)
	Parasites	*Leishmania donovani* and *Leishmania infantum*	0.05, 1.5 and 10 μM	*Leishmania donovani* and *Leishmania infantum* ↓	Corral et al. (2014a)
	Parasites	*Leishmania infantum*	1 or 5 mg/kg/day	*Leishmania infantum* ↓	Corral et al. (2014b)
	Bacteria	*S. aureus*	900 μg/mL	*S. Aureus* ↓	Pérez-Köhler et al. (2015)
	Bacteria	*Pasteurella multocida*	50 mg/kg/day	*Pasteurella multocida* ↓	Alam et al. (2018)
	Bacteria	*H. pylori*	500 mg/L or 100 mg/L	*H. Pylori* ↓	Jonkers et al. (1999b)
	Bacteria	Vancomycin-resistant *Enterococci*	8–16 μg/mL	Vancomycin-resistant *Enterococci* ↓	Jonkers et al. (1999a)
	Bacteria	Methicillin-resistant *Staphylococcus aureus*	0.1–9.6 μg/mL	*Methicillin*-resistant *Staphylococcus aureus* ↓	Sharifi-Rad et al. (2014)
	Fungus	*T. rubrum*	0.04 mg/mL	*T. Rubrum* ↓	Robles-Martínez et al. (2019)
Antidiabetic activity	—	Sprague-Dawley rats	15, 30, and 45 mg/kg/day	Blood glucose, FBG, TG, BUN, sCr, collagen I, TGF-β1, and p-ERK1/2 ↓	Huang et al. (2017a)
	—	Wistar rats	16 mg/kg/day	Blood glucose, IL-1β, IL-6, NF-κB, and TGF-β1 ↓ Iκβ ↑	Arellano Buendía et al. (2018)
	—	Wistar rats	16 mg/kg/day	CTGF, α-SMA, TGF-β1, TAS, Nrf2/Keap1 ratio, HIF-1α, VEGF ↓ Nephrin, KIM-1, Epo, Epo-R ↑	Arellano-Buendía et al. (2020)
	—	C57BL/6J mice, human umbilical vein endothelial cells	30 mg/kg/day and 10 μg/mL	TNF-α, VCAM-1, iNOS, MMP-2, MCP-1, and NF-κB, cleaved-caspase3 activity, Bax/Bcl2 ratio, Nrf2,VCAM-1, iNOS, MMP-2, MCP-1, and NF-κB ↓ Cell proliferation, Nrf2 ↑	Li et al. (2020)
	—	Sprague− Dawley rats	8 and 16 mg/kg	Blood glucose, ICA level ↓ Improved islet degeneration, necrosis and shrinkage ↑	Osman et al. (2012)
	—	Wistar rats	4, 8, and 16 mg/kg	Blood glucose, arrhythmia score ↓	Huang et al. (2013)
	—	Wistar albino rats	37.5 μg/mL	Neutrophil, mononuclear cell, intraepithelial edema, and dermal edema density ↓ Fibroblast, angiogenesis and collagen density ↑	Toygar et al. (2020)
Cardioprotective activity	—	Wistar albino rats	9 mg/kg/day	CF, TNFα, IL-1β, IL-6, cTnI, cTnT, LDH ↓ SOD3, GPX1, CAT ↑	Mousa et al. (2022)
	—	C57BL/6 J mice	10 mg/kg/day	TMA, TMAO, and γBB levels ↓	Panyod et al. (2022)
	—	Spontaneous hypertension rats	14 mg/kg/day	PCNA, α-SMA, IL-1β, IL-6, TNF-α, NLRP3 ↓	Liu et al. (2022)
	—	Sprague-Dawley rats	1.2, 1.8, 3.6 mg/kg/day	MDA, iNOS, p-eNOS, p-JNK, Cyt-c, Caspase-3, Caspase-9 ↓ SOD, CAT, GSH-Px, eNOS ↑	Xu et al. (2020)
	—	C57BL/6 J mice	18.2 mg/kg/day	Caspase-3 ↓	Cai et al. (2019a)
	—	Sprague-Dawley rats	7, 14 mg/kg/day	Myocardial enzyme levels ↓	Cui et al. (2021)
	—	Wistar rats	5, 10, 20 mg/kg/day	PECAM-1, Ang-2, PDGFR-β ↑ Caspase-3, RIP3 ↓	Shi et al. (2018)
	—	Wistar rats	5, 10, 20 mg/kg/day	BNP, LC3-II, Beclin-1, and β-MHC ↓ p-Akt, p-PI3K, and p-ERK ↑	Ba et al. (2019)
	—	Vascular endothelial cells	0, 25, 50, 100 mg/L	PCNA, BCL-2, JAK2, STAT3 ↓	Sun et al. (2021b)
	—	HepG2 cells	100 and 200 μM	HNF1α, PCSK9, SREBP2 ↓	Nawaka et al. (2022)
Hepatoprotective activity	—	Swiss mice	10 mg/kg/d	NLRP3, Caspase-1, IL-1β↓ Bcl-2, Ki-67 ↑	Samra et al. (2020)
	—	RAW264.7 cells Balb/c mice	40、20、10 mg/kg/d	IL-1β, IL-6, MDA, Caspase-3, Caspase-8, BAX, NQO1 ↓	Gong et al. (2024)
	—	Rabbits	100 and 200 mg/kg	TNF-α ↓	El-Gindy et al. (2023)
	—	C57BL/6 mice	5 and 20 mg/kg/day	AST, ALT, CYP2E1, TNF-α, IL-1β, IL-6, SREBP-1 ↓	Panyod et al. (2016)
	—	C57BL/6 mice	3、10、30 mg/kg	TNF-α, IL-1β ↓	Li et al. (2022b)
	—	Kupffer cells Sprague Dawley rats	3.75, 7.5, 15 μM	p-IRE, p-ASK, TRAF2, xbp −1, IL-1β, IL-18, IL-6, TNF-α ↓	Nan et al. (2021)
	—	BRL-3A cells	3.75, 7.5, 15, 30 μM	KGF, Gadd45a, c-Fos, Dusp5, Phospholipase A2 ↓	Hong et al. (2019)
	—	Wister albino rats	45 mg/kg	Caspase-3 ↓	Orabi et al. (2020)
	—	Wistar rats	30 mg/kg	NF-κB ↓ Heme oxygenase 1, KLF9 ↑	Yang et al. (2017)
	—	Balb/c male mice	8–20 mg/kg/day	VCAM-1, IAM-1, iNOS ↓	Bruck et al. (2005)
	—	bovine hepatic cells	10, 20, 30, 40, 50, 60 μM	Nrf2 ↑	Jin et al. (2024)
Neuroprotective activity	—	C57Bl/6 mice	180 mg/kg/day	Aging-induced cognitive dysfunction ↓	Li et al. (2012b)
	—	Rats	1, 10, 50 mg/kg	Traumatic brain injury ↓	Chen et al. (2014)
	—	Sprague-dawley rats	180 mg/kg/day	Endoplasmic reticulum stress-related cognitive deficits ↓	Zhu et al. (2015)
	—	SD rats	50 mg/kg	Ischemic stroke ↓	Lin et al. (2015)
	—	Sprague-dawley rats	2,10,50 mg/kg	The recovery of motor function after acute traumatic spinal cord injury ↑	Lv et al. (2015)
	—	Sprague-dawley rats	180 mg/kg/day	The cognitive deficits caused by tunicamycin ↓	Xiang et al. (2017)
	—	mice	10 mg/kg/day	The cognitive function ↑	Zhang et al. (2018)
	—	C57Bl/6J mice	2, 10, 50 mg/kg	Chronic social defeat stress ↓	Gao et al. (2019)
	—	Sprague-dawley rats	30, 70 mg/kg	Brain edema and blood-brain barrier dysfunction ↓	Shao et al. (2019)
Anticancer activity	Breast cancer	MCF-7 cells	—	The spread of cancer cells ↓	Hirsch et al. (2000)
	Gastric cancer	MGC-803 cells sGC-7901 cells	—	The growth of cancer cells ↓	Ha and Yuan, (2004)
	—	THP-1 cells	—	The proliferation of cells ↓	Liao et al. (2009)
	Colon cancer	MEC-1 cells	—	The growth of cells ↓	Wu et al. (2015)
	Colon cancer	HCT-116, LS174T, HT-29, Caco-2 cells	6.2–310 μM	Apoptosis ↑	Bat-Chen et al. (2010)
	Brain Cancer	U87MG cells	10, 30, 60, 90 μM	The survival of tumor cells ↓	Cha et al. (2012)
	Liver cancer	HepG2 cells	35 μM	The viability of cancer cells ↓	Chu et al. (2012)
	Liver cancer	HepG2 cells	0, 15, 20, 25, 35, 40, 50 μM	Autophagy ↑ The viability of cancer cells ↓	Chu et al. (2013)
	Bone cancer	MG-63, U20S, 143-B, SaOS-2, HOS cells	5, 7.5, 10 μM	The viability of cancer cells ↓	Jiang et al. (2013)
	Gastric cancer	MGC-803, BGC-823, sGC-7901 cells	0.1, 1, 10 μg/mL	Apoptosis ↑ The proliferation of cancer cells ↓	Zhang et al. (2015b)
	Pancreatic cancer	MIA PaCa-2, HepG2, OAW42, MCF-7 cells	20, 40, 60, 80, 100, 120, 140, 180, 200 µM	The proliferation of cancer cells ↓	Chhabria et al. (2015)
	Brain Cancer	SK-N-SH cells	0, 0.5, 1, 5, 10, and 20 μmol/L	The growth of cancer cells ↓	Zhuang et al. (2016)
	Liver cancer	SK-HEP-1 and BEL-7402 cells Athymic nude mice	0, 1, 2, 4, 8, 10, 16, 20, 32, 40, and 64 μg/mL; 0, 3, 6, and 10 μg/mL; 5 mg/kg/day every 2 days	The growth of cancer cells ↓	Zou et al. (2016)
	Brain Cancer	U251 cells	15, 30, 60, and 90 μg/mL	The proliferation and colony formation ↓	Li et al. (2018)
	Colon cancer	HCT116 cells	25 µM	Apoptosis ↑ The survival and proliferation of cancer cells ↓	Li et al. (2019a)
	Gastric cancer	Patients with progressive gastric adenocarcinoma	\	Apoptosis ↑ The proliferation of cancer cells ↓	Zhang Z. D. et al. (2008)
	Breast cancer	HCC-70 cells	1 μM, 3 μM, 10 μM, 12 μM, 20 μM, and 45 μM	Apoptosis ↑ The growth of cancer cells ↓	Rosas-González et al. (2020)
	Lung cancer	A549 cells and NCI-H460 cells	10, 20, 40, 60 μg/mL	Apoptosis ↑ The proliferation of cancer cells ↓	Pandey et al. (2020)
	Renal cancer	RCC-9863 cells	0.016 mg/mL, 0.05 mg/mL, 0.1 mg/mL	Apoptosis ↑ The viability of cancer cells ↓	Song et al. (2015)
	Ovarian cancer	SKOV3 cells	25 ug/mL	Apoptosis ↑	Xu et al. (2014)

### Application of allicin

**FIGURE 2:**
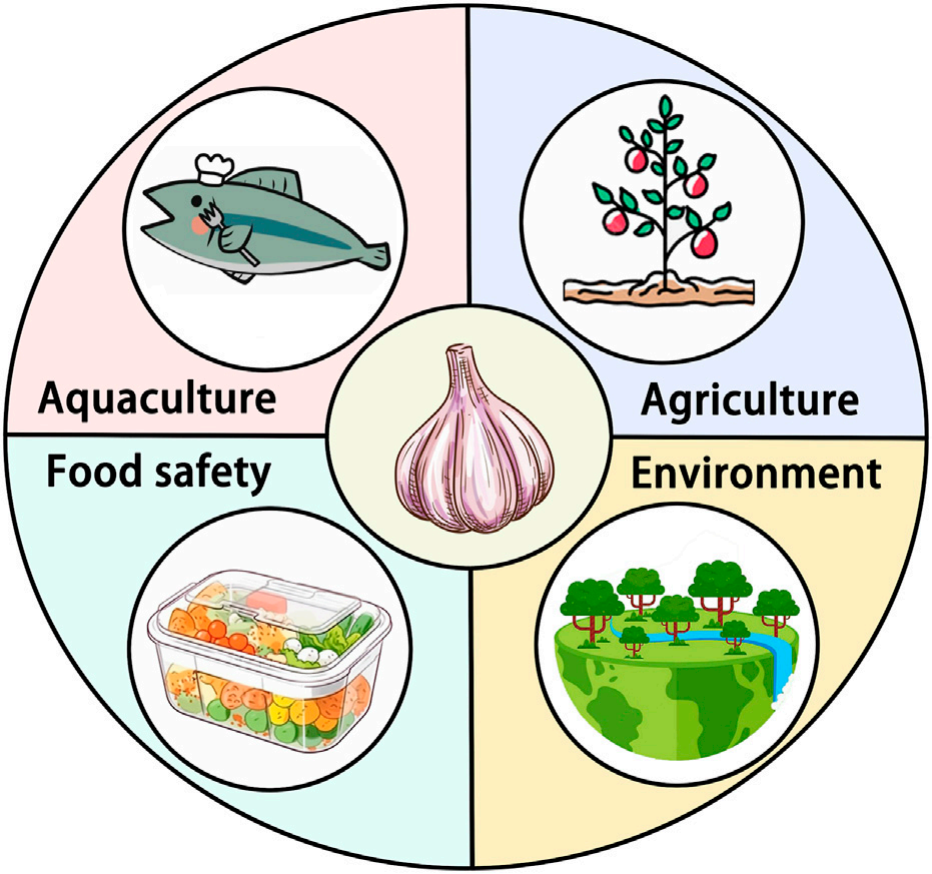
The application of allicin.

Numerous studies have also shown that allicin plays an important role in aquaculture, agriculture, and animal husbandry (Figure 2). On the one hand, allicin can prevent ascaris infection and exterminate poultry red mites in chickens (Kang et al., 2023; Velkers et al., 2011). After allicin was added to the hens’ diet, their offspring showed better immunity and growth performance in early life (Gong et al., 2020). On the other hand, allicin can improve the gastrointestinal tract development of piglets (Tatara et al., 2008). After allicin was added to the pregnant sows’ diet, allicin can enhance sow reproductive performance and placental angiogenesis (Peng et al., 2024). In addition, allicin can also be used as a feed additive to improve the immunity of rabbits and the growth of large yellow croaker larvae (Abu El Hammed et al., 2016; Huang W. et al., 2020). The fatty liver in fish is a main problem in aquaculture. In aquaculture, allicin can improve fatty liver caused by environmental estrogen (Zhang et al., 2022). Methane is the main component of natural gas, and it is also an important greenhouse gas in the atmosphere. Ruminants are thought to be a significant contributor to methane emissions. Studies have shown that supplementary allicin of ruminant feed can reduce the population of methanogens (Ma et al., 2016). This is good for the environment and has potential economic benefits. Interestingly, allicin not only enhanced the volatile fatty acid yield during sludge fermentation but also increased the shelf life of bullfrogs during refrigeration (Lan et al., 2023; Wang F. et al., 2022). These findings indicate that allicin has good application prospects in aquaculture, agriculture, and animal husbandry. As a carcinogenic metabolite, acrylamide is formed during food heating. Some studies have shown that acrylamide not only causes liver damage but also damages the intestinal barrier (Yuan et al., 2021; Zhang et al., 2012). Therefore, reducing acrylamide content is important to protect public health. Adding a proper amount of allicin can ensure food safety by reducing acrylamide content during food processing (Li X. et al., 2022). In a word, allicin has broad application prospects. In the future, exploring the application of allicin could greatly improve our lives.

## Conclusion and prospective

Garlic has been a popular plant since ancient times. In this review, we summarized the health benefits and applications of allicin. Based on the reviewed literature, the current body of research on allicin faces several significant limitations that constrain a full understanding of its therapeutic potential. A primary constraint is the heavy reliance on pre-clinical evidence from *in vitro* and animal studies, with a conspicuous lack of validation from robust human clinical trials. This pre-clinical focus is compounded by fundamental challenges related to allicin’s inherent physicochemical properties, including its chemical instability, strong odor, and low bioavailability, which collectively hinder its clinical application. Furthermore, inconsistencies in reported results across different studies, potentially due to variations in experimental models and a lack of standardized protocols, further obscure a clear consensus on its efficacy and mechanisms.

To address these limitations and advance the field, future research efforts must prioritize several key directions. The most critical step is to transition from pre-clinical models to well-designed human clinical trials to conclusively establish efficacy, safety, and appropriate dosing for various health conditions. Concurrently, substantial research investment is needed into developing advanced formulation strategies, such as nanoparticle-based delivery systems, to overcome the challenges of stability, bioavailability, and patient compliance. The promising area of drug synergy, where allicin enhances the effects of conventional antibiotics and anticancer agents, warrants expanded exploration as a strategy to combat drug resistance. Ultimately, the establishment of standardized protocols for allicin preparation and biological testing is a fundamental prerequisite for ensuring the reproducibility and reliability of future research, paving the way for its potential translation into validated clinical and nutraceutical applications.
